# Genes Regulate Blood Pressure, but “Environments” Cause Hypertension

**DOI:** 10.3389/fgene.2020.580443

**Published:** 2020-11-09

**Authors:** Lin-dan Ji, Nelson L. S. Tang, Zhi-feng Xu, Jin Xu

**Affiliations:** ^1^Department of Biochemistry, School of Medicine, Ningbo University, Ningbo, China; ^2^Zhejiang Key Laboratory of Pathophysiology, Medical School of Ningbo University, Ningbo, China; ^3^Department of Chemical Pathology, Faculty of Medicine, The Chinese University of Hong Kong, Hong Kong, China; ^4^Laboratory for Genetics of Disease Susceptibility, Li Ka Shing Institute of Health Sciences, The Chinese University of Hong Kong, Hong Kong, China; ^5^Department of Cardiology, Ningbo No. 7 Hospital, Ningbo, China; ^6^Department of Preventive Medicine, School of Medicine, Ningbo University, Ningbo, China

**Keywords:** hypertension, blood pressure, single nucleotide polymorphism, environment, gene

## Introduction

Hypertension is among the first traits studied using genome-wide association study (GWAS). At first, GWAS by the Well-come Trust Case Control Consortium in 2007 did not identify any genome-wide significant single nucleotide polymorphism (SNP) (The Wellcome Trust Case Control Consortium, 2007), however, it was still asserted that GWAS would open the door to find the “missing heritability” of hypertension. In the following years, GWASs of hypertension were still unsuccessful in identifying robust loci until 2009. After shifting attention to the underlying quantitative traits (systolic blood pressure and diastolic blood pressure) and application of GWA meta-analyses of multiple cohorts to enlarge the sample size, the Global Blood Pressure Genetics (Global BPgen) Consortium and the Cohorts for Heart and Aging Research in Genome Epidemiology (CHARGE) Consortium identified 13 genome-wide significant signals (Levy et al., 2009; Newton-Cheh et al., 2009), although these SNPs had very small effect sizes and accounted for <0.2% of the overall blood pressure variation in the study populations.

## Will GWAS Unlock the Genetic Basis of Hypertension?

There was a heated debate in *HYPERTENSION* on December 2010. Dominiczak and Munroe described the successes of aforementioned two studies, and predicted a bright future for GWAS in hypertension (Dominiczak and Munroe, 2010). On the contrary, Kurtz contended that GWAS had failed, and would continue to fail, to delineate the genetic basis of hypertension. He suggested that efforts and dollars should be shifted to other strategies and technologies that may hold greater chance for advancing our understanding of the genetic etiology of hypertension (Kurtz, 2010). Despite different opinions, both pro and con sides shared some common ground: low-frequency/rare variants are important, and the sample size should be further enlarged.

In 2016, three large-scale GWASs of hypertension were published in *NATURE GENETICS* (Ehret et al., 2016; Liu et al., 2016; Surendran et al., 2016). After applying newly designed microarray chips and meta-analyses of multiethnic populations with unprecedented large sample sizes (>300,000), these studies identified several new common loci of modest effects, and provided insights into the impact of low-frequency/rare variants. Subsequently, other genome-wide analyses of blood pressure traits (systolic, diastolic, and pulse pressure) were carried out in people of European ancestry drawn from UK Biobank (UKB), the International Consortium of Blood Pressure Genome Wide Association Studies (ICBP), the Genetic Epidemiology Research on Adult Health and Aging (GERA) cohort, and other combined cohorts with very large sample size (Hoffmann et al., 2017; Warren et al., 2017; Evangelou et al., 2018). These studies identified hundreds of novel blood pressure loci that offer new biological insights into blood pressure regulation. Also, trans-ethnic genome-wide association study of blood pressure in up to 776,078 participants from the Million Veteran Program (MVP) and collaborating studies discovered 208 novel common blood pressure SNPs and 53 rare variants (Giri et al., 2019). Table 1 listed all high quality GWASs for hypertension or blood pressure published in NATURE GENETICS since 2007.

**Table 1:** GWASs for hypertension or blood pressure published in NATURE GENETICS since 2007.

**PMID**	**Year**	**Disease/trait**	**Initial sample size**	**Replication sample size**	**Major finding**
19430479	2009	DBP, SBP, HT	29,136 European ancestry individuals	34,433 European ancestry individuals	They identified 13 SNPs for SBP, 20 for DBP, and 10 for hypertension.
19430483	2009	DBP, SBP	34,433 European ancestry individuals	Up to 100,347 European ancestry individuals, up to 12,889 Indian Asian ancestry individuals	They identified association between systolic or diastolic blood pressure and common variants in eight regions near the CYP17A1, CYP1A2, FGF5, SH2B3, MTHFR, c10orf107, ZNF652, and PLCD3 genes.
21572416	2011	BP	19,608 East Asian ancestry individuals	30,765 East Asian ancestry individuals	They identified genome-wide significant associations with SBP or DBP, which included variants at four new loci (ST7L-CAPZA1, FIGN-GRB14, ENPEP, and NPR3) and a newly discovered variant near TBX3.
21909110	2011	PP	74,064 European ancestry individuals	48,607 European ancestry individuals	They identified at genome-wide significance four new PP loci (at 4q12 near CHIC2, 7q22.3 near PIK3CG, 8q24.12 in NOV and 11q24.3 near ADAMTS8), two new MAP loci (3p21.31 in MAP4 and 10q25.3 near ADRB1) and one locus associated with both of these traits (2q24.3 near FIGN).
26390057	2015	DBP, SBP, PP	31,516 East Asian ancestry individuals, 35,352 European ancestry individuals, 33,126 South Asian ancestry individuals	87,205 individuals, 48,268 East Asian ancestry individuals, 68,456 European ancestry individuals, 16,328 South Asian ancestry individuals	They identified genetic variants at 12 new loci to be associated with blood pressure.
27618447	2016	DBP, SBP, PP, HT	Up to 165,276 European ancestry individuals, up to 27,487 South Asian ancestry individuals	Up to 125,713 European ancestry individuals, up to 2,641 South Asian ancestry individuals, 4,632 Hispanic individuals, 22,077 African American individuals	They identified 30 new blood pressure- or hypertension-associated genetic regions in the general population, including 3 rare missense variants in RBM47, COL21A1 and RRAS with larger effects than common variants.
27618448	2016	DBP, SBP, PP, HT	120,473 European ancestry individuals, 21,503 African American individuals, 4,586 Hispanic individuals	154,543 European ancestry individuals, 26,183 South Asian ancestry individuals	They identified 31 new blood pressure-associated loci.
27618452	2016	DBP, SBP	Up to 201,529 European ancestry individuals	Up to 140,886 European ancestry individuals	They identified 66 blood pressure-associated loci, of which 17 were new; 15 harbored multiple distinct association signals.
27841878	2016	DBP, SBP, PP	295,529 European ancestry individuals, 8,231 Latino individuals, 3,058 African American individuals, 2,029 African British individuals, 7,701 East Asian ancestry individuals, 2,735 South Asian ancestry individuals, 1,979 mixed and unknown ancestry individuals	NA	They identified 39 new loci among 75 genome-wide significant loci.
28135244	2017	DBP, SBP, PP	140,886 European ancestry individuals	190,318 European ancestry individuals	They identified 107 blood pressure-associated loci.
29403010	2018	DBP, SBP, PP	136,615 Japanese ancestry individuals	NA	They identified 1,407 trait-associated loci, 679 of which were novel.
30224653	2018	DBP, SBP, PP	757,601 European ancestry individuals	249,262 European ancestry individuals	They identify 535 novel blood pressure loci.
30578418	2019	DBP, SBP, PP	365,998 European ancestry individuals, 63,490 African ancestry individuals, 22,802 Hispanic individuals, 4,792 Asian ancestry individuals, 2,695 Native American ancestry individuals	299,024 European ancestry individuals, 17,277 individuals	They discovered 208 novel common blood pressure SNPs and 53 rare variants.

*BP, blood pressure; DBP, diastolic blood pressure; SBP, systolic blood pressure; PP, pulmonary pressure; HT, hypertension*.

## Gene–Environment Interplay

Thirteen years GWAS for hypertension, from the beginning “failure” to the recent “success,” can we say GWASs have deciphered the genetic architecture of hypertension? The answer is obviously NO. This is because of the fact that environmental factors and gene-environment interactions are likely major contributors to the development of hypertension, however, they were largely ignored in present GWASs (Cooper, 2018).

As widely accepted, hypertension is a consequence of significant interaction between genetic and environmental factors. Here, the meaning of “environment” is extensive, including intrauterine, postnatal and evolutionary environments. It has long been known that intrauterine environmental factors (e.g., maternal nutritional perturbation, toxin exposure, and stress during pregnancy) may result in hypertension in adult life. Recent epidemiological and experimental studies further indicated that paternal environmental factors, before conception and during sperm development, were also linked to the development of hypertension in later life (Li et al., 2016). On the other hand, classic epidemiological studies have addressed many postnatal environmental risk factors for hypertension, including living environments (cold temperature, air pollution, and toxins), life styles (lack of physical activity, psychological stress, smoking, alcohol abuse, drug use, improper nutrition, excessive salt intake, and obesity), and other demographic differences in age, gender, race, socioeconomic status, etc. From an evolutionary perspective, hypertension can be viewed as a maladaptation disease caused by the discrepancy between today's lifestyles and ancient adaptive genotypes. Physiologically, many risk factors for hypertension, such as enhanced salt and water avidity and vascular contractility, were adaptive traits associated with salt scarcity in the hot and humid climate of the ancestral African environment. As humans migrated out of Africa to cooler environments, the originally selected genes became maladaptive for new environments and turned into risk factors for hypertension (Young et al., 2005; Ji et al., 2016).

## Discussion

Due to the complexity of essential hypertension, previous GWAS didn't find many robust variants. If more related factors are considered, and method like phenomics is used, future studies may help us to deeply understand this complex disease. As emphasized by Jeremy Berg, editor-in-chief of SCIENCE, genes alone do not determine our futures—environmental factors and chance also play important roles (Berg, 2016). Indeed, 13 years GWASs for hypertension really made a big step forward, however, if we leave behind another important factor, the environment, can also impede our progress. Incredibly, the environmental effects were ignored in almost all published GWASs of blood pressure traits or hypertension. Some studies were conducted in well-established cohorts like Global BPGen and CHARGE, and many environmental data should be available. Although analyzing the gene-environment interaction is still a great challenge, GWASs without consideration of environmental effects will definitely not go to address the key question regarding the genetic architecture of hypertension.

## Author Contributions

L-dJ and JX conceived the opinion. L-dJ, NT, Z-fX, and JX wrote the manuscript. All authors contributed to the article and approved the submitted version.

## Conflict of Interest

The authors declare that the research was conducted in the absence of any commercial or financial relationships that could be construed as a potential conflict of interest.
